# Potential for Cost Recovery: Women’s Willingness to Pay for Injectable Contraceptives in Tigray, Ethiopia

**DOI:** 10.1371/journal.pone.0064032

**Published:** 2013-05-20

**Authors:** Ndola Prata, Suzanne Bell, Karen Weidert, Amanuel Gessessew

**Affiliations:** 1 Bixby Center for Population, Health and Sustainability, School of Public Health, University of California, Berkeley, California, United States of America; 2 Department of Gynaecology and Obstetrics, Mekele University College of Health Sciences, Mekele, Ethiopia; Indiana University, United States of America

## Abstract

**Objective:**

To investigate factors associated with a woman’s willingness to pay (WTP) for injectable contraceptives in Tigray, Ethiopia.

**Methods:**

We used a multistage random sampling design to generate a representative sample of reproductive age women from the Central Zone of Tigray, Ethiopia to participate in a survey (N = 1490). Respondents who had ever used injectable contraceptives or who were interested in using them were asked whether they would be willing to pay, and if so, how much. Logistic regression odds ratios (ORs) with 95% confidence intervals (CIs) and p-values were used to assess which factors were associated with WTP in our final model.

**Findings:**

On average, respondents were willing to pay 11 birr ($0.65 USD) per injection. Being married, completing any amount of education, having given birth, and having visited a health facility in the last 12 months (whether received family planning information or not) were associated with statistically significantly increased odds of WTP. Having initiated sexual activity and having 1–2 children (compared to 0 children) were associated with statistically significantly decreased odds of WTP. We also detected two significant interactions. Among women who prefer injectable contraceptives, their odds of WTP for injectable contraceptives vary across length of time they have used them. And among women who work for pay, their odds of WTP for injectable contraceptives vary by whether they agree with their husband/partner about the ideal number of children.

**Conclusion:**

In a sector that continually struggles with funding, cost recovery for contraceptive services may offer a means of improved financial sustainability while increasing rural access to injectable contraceptives. Results indicate there are opportunities for cost recovery in rural Tigray, Ethiopia and highlight factors that could be leveraged to increase WTP for injectable contraceptives.

## Introduction

The fundamental role of contraception in improving maternal and child health is increasingly recognized by policy-makers, researchers, and donors alike. Ahmed et al. (2012) reported that 44% of potential maternal deaths worldwide were averted by contraceptive use in 2008. This is equivalent to 38 maternal deaths prevented for every 100,000 reproductive age women using contraceptive methods every year [1]. Cleland et al. (2012) concluded that an additional 30% of maternal deaths could be averted by fulfilling unmet need for contraception in developing countries, particularly in sub-Saharan Africa where unmet need for family planning, and consequently maternal mortality, is high [2].

The numerous benefits of family planning are clear. Yet current donor funding and government expenditures on reproductive health services in sub-Saharan Africa are not sufficient [3], and therefore, the issue of financial stability is a main concern in the development and implementation of family planning programs [4], [5]. As governments and providers investigate options for cost recovery and revenue generation, it is critical to understand factors associated with women’s willingness to pay (WTP) for contraceptives.

In Ethiopia, the total fertility rate (TFR) has declined from 5.4 children per woman in 2005 to 4.8 children per woman in 2011 [6], [7]. However, the 28% unmet need for family planning in rural areas and the desired family size of 4.3 children per woman highlight the potential for further decline in TFR by meeting demand for contraception [6]. The growing use of modern contraceptives and declining TFR in Ethiopia is largely attributed to the dramatic rise in use of injectable contraceptives, which increased from 3% to 21% among married women between 2000 and 2011 [6], [8]. This growth is not surprising given the 2005 Demographic Health Survey (DHS) finding that 72% of women reported a preference for injectable contraceptives [7]. Injectable contraceptives are currently used by 14% of married Ethiopian women, with implants and pills being the second and third most commonly-used methods at 2.3% and 1.5%, respectively [9]. However, access to injectable contraceptives is not universal and disparities exist in the country. In 2011, 18% of women in rural Ethiopian communities were currently using injectable contraceptives compared to 35% of women in urban communities despite similar levels of preference [6]. In rural areas, the only source of injectable contraceptives is government facilities (i.e. hospitals, health centers, or most commonly, health posts). Women receive injectable contraceptives for free from these facilities, but women often live far from these facilities or arrive at facilities where the providers are not present, have many clients whom they are treating, or do not have any injectable contraceptives in stock.

Public health programs are increasingly charging user fees to improve long-term sustainability in an attempt to strike a balance between cost recovery and program reach [4]. WTP for services among current and prospective clients influences the opportunity for cost recovery [10], [11], [12]. Studies assessing WTP and the impact of price increases on demand for health services and commodities vary in their findings, but generally conclude that assigning context-dependent user fees is acceptable and will result in limited impact on demand [11], [12], [13], [14], [15], [16], [17]. Consensus has not been reached on whether price increases result in non-differential price responsiveness of different income groups [13], [14]. However, research in resource poor countries has suggested that people living in poverty are willing to pay for services they value, such as family planning, and perceive as high quality [13], [17], [18]. One study in Egypt found that 45% of women surveyed were willing to pay for injectable contraceptives [19]. More research is needed to build consensus around the impact of price increases on demand and to determine the factors that impact one’s WTP for specific services or commodities. Assessing women’s WTP for injectable contraceptives and the related factors influencing their WTP can provide program planners and policy-makers with critical information.

The objective of this paper is to explore factors associated with women’s willingness to pay and the amount women are willing to pay for injectable contraceptives in rural Ethiopia.

## Methods

Human subjects approval was obtained from the Center for Protection of Human Subjects (CPHS) at the University of California Berkeley (CPHS Protocol ID 2011/07/3465). We used a multi-stage random sampling design, which provides representative data for the Central Zone of Tigray, Ethiopia. All women of reproductive age (i.e. those between 15 and 49 years of age) in the households randomly selected from the randomly selected *kebeles* (villages) were eligible to participate in the study.

A total of sixteen trained interviewers and three supervisors were sent to the three selected *woredas* (districts) in teams of five to six interviewers and one supervisor. Data collection took a total of 15 days. Our response rate was 99%, resulting in 1490 respondents, all of whom provided verbal informed consent.

The survey data serves as the baseline for a larger evaluation of an ongoing project testing the combination of social marketing and community based distribution (CBD) of injectable contraceptives in Tigray, Ethiopia. Tigray is the northernmost region of Ethiopia and is a predominantly rural area. Conducted in October 2011, the survey drew from the demographic, fertility, and family planning sections of the Demographic and Health Survey (DHS) and included additional questions regarding injectable contraceptives and previous payment/WTP. Among women surveyed, those who had ever used injectable contraceptives or expressed interest in using injectable contraceptives were asked the WTP questions. The first question elicited a dichotomous yes/no response to whether the woman would be willing to pay for injectable contraceptives. The follow-up question, if she responded yes, was open-ended and inquired how much she would be willing to pay for injectable contraceptives in birr (the local currency). At the time of the survey, 1 USD was equivalent to 17 Ethiopian birr. For further details, see the Baseline Survey Report [20].

**Figure 1:**
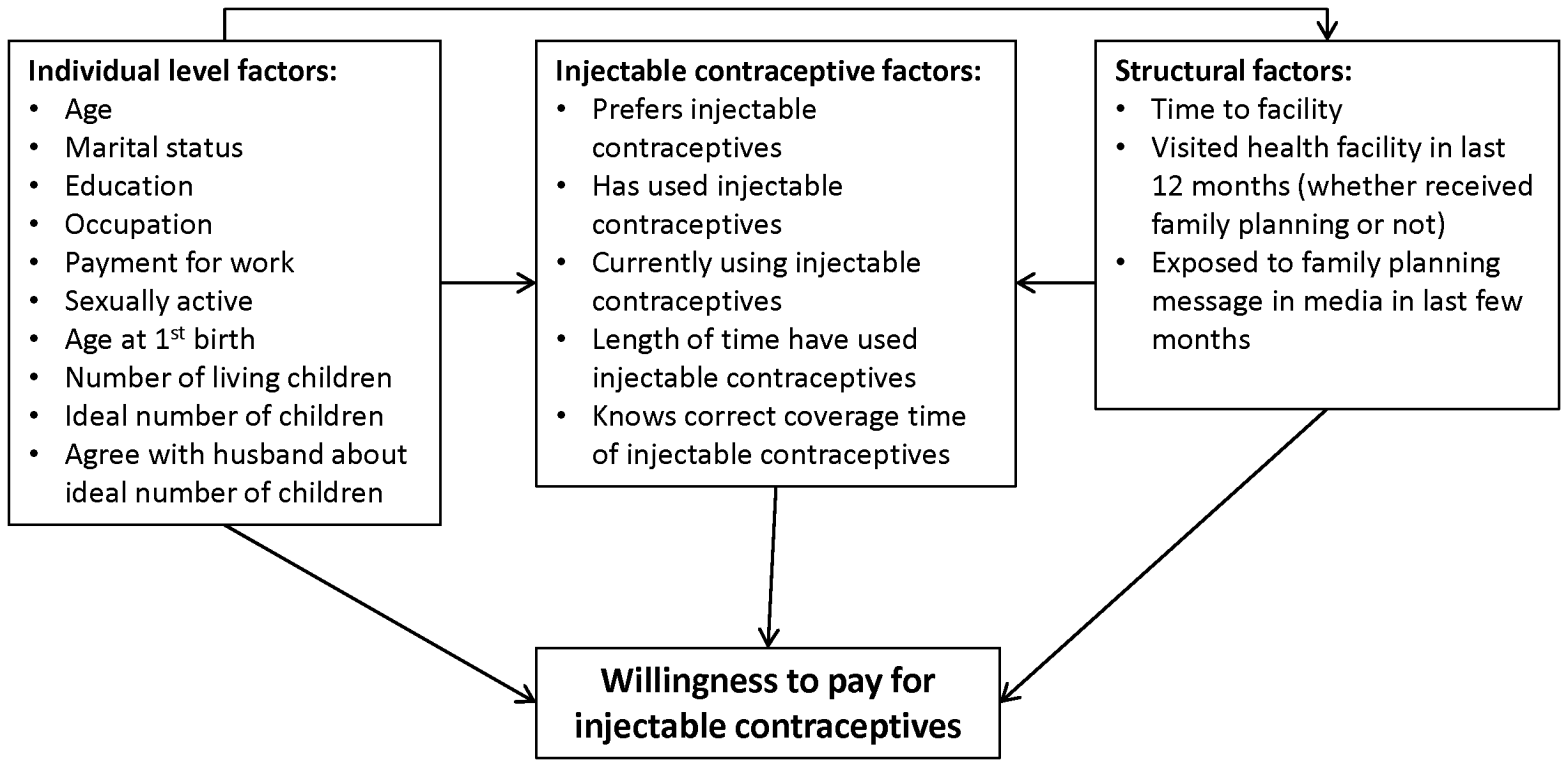
Factors considered to be associated with willingness to pay for injectable contraceptives that were available for analysis.

Figure 1 presents factors considered to be associated with WTP that were available for analysis. These were categorized as individual level, injectable contraceptive, or structural factors. ‘Individual level factors’ are those related to a woman’s social status, economic status, or reproductive history; ‘injectable contraceptive factors’ are those related to injectable contraceptive use, preference, or knowledge; and ‘structural factors’ are those external to the individual surveyed but still related to health care utilization and knowledge, e.g. distance to the nearest facility (estimated by respondent in hours and minutes), having visited a facility in the last year, and exposure to family planning messages on the television, radio, or in newspapers in the last few months.

In building our models, we began by using results from the bivariate analysis to determine which factors to include in the multivariate logistic regression. All covariates with p≤0.05 were included in the model. We then removed all covariates that were not significant at the p≤0.20 level in the multivariable model. For groups of covariate categories (e.g. marriage categories, education categories, etc.), we used the Wald test to determine whether their contribution to the explanatory power of the model was significant, retaining all covariate categories with p≤0.20. We kept age in the model despite it not being significant because we hypothesized it to be a confounder of other relationships. We tested the possibility of effect measure modification between receiving payment for work and agreement with husband/partner’s ideal number of children, as well as between preferred contraceptive and length of time using injectable contraceptives; a Wald test revealed that both sets of cross-products were significant at the p≤0.05 level.

Our final model included age, marital status, education, payment for work, whether respondent has had sex, age at first birth, number of living children, whether respondent agrees with husband/partner about ideal number of children (those not in a relationship were categorized as ‘don’t know/not with partner’), preferred method of contraception (injectable contraceptive versus other), total time using injectable contraceptives, health facility visit in last 12 months (whether received family planning information or not), and all cross-products associated with the aforementioned interactions. Based on our multi-stage random sampling design, we used Stata’s vce(cluster *varname*) option in all logistic regressions to obtain a robust variance estimate that adjusts for within cluster correlation [21]. Logistic regression odds ratios (ORs) with 95% confidence intervals (CIs) and p-values were used to assess which factors were associated with WTP; the requirement for statistical significance was a p-value≤0.05 and a 95% CI that did not cross 1.0. Bonferroni p-value adjustments for multiple comparison were performed to account for the 7 hypotheses tested related to the interactions [21]. We used how much women were willing to pay to create a WTP-based demand curve. All analyses were conducted using Stata/IC version 11.2 [21].

## Results

**Figure 2:**
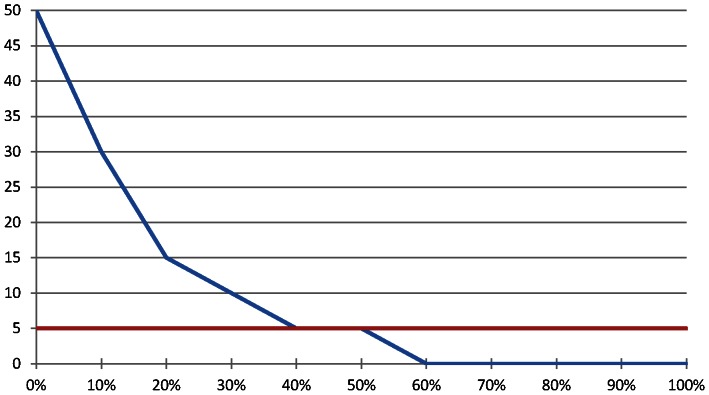
Willingness to pay for injectable contraceptives among women of reproductive age who have ever used them or who are interested in using them (N = 767)*. *Approximately 3% of women were willing to pay between 50 and 200 birr; we have only labeled up to 50 birr for visual purposes X-axis: Percentage of women Y-axis: Amount Willing to Pay (in Ethiopian birr: 1 USD = 17 birr) Red line: 5 birr: cost of injectable contraceptives in project.

Among the 1490 women surveyed, 1013 (68%) had ever used injectable contraceptives or expressed interest in using injectable contraceptives and thus were asked the WTP questions. Overall, 68% of these women were willing to pay for injectable contraceptives. The open-ended WTP question revealed that women were willing to pay, on average, 11 birr ($0.65 USD), and that 52% of women were willing to pay 5 birr for injectable contraceptives; 5 birr is the current cost of one injection in the ongoing project (Figure 2).

As seen in Tables 1, 2, and 3, there were not marked differences between the full sample and the subpopulation who were asked the WTP questions, with the exception of ever use of injectable contraceptives and preferred method of contraception. Among women from the subpopulation in our analyses, 67% had ever used injectable contraceptives and 77% said it was their preferred method (Table 2).

**Table 1:** Individual level factors among all women surveyed and among the WTP study subpopulation (i.e. women who had ever used injectable contraceptives or expressed interest in using them).

		Full Sample N = 1490*		Subpopulation N = 1013	
		%	N	%	N
Age					
	15–19	19.2	286	15.1	151
	20–24	16.9	252	20.0	200
	25–29	17.9	267	22.7	227
	30–34	15.7	234	18.4	184
	35–39	13.4	199	13.9	139
	40–44	8.1	121	6.5	65
	45–49	7.5	112	3.5	35
Marital status					
	Never married	13.6	202	8.3	84
	Married/cohabiting	72.3	1077	81.0	820
	Divorced/widowed	13.9	207	10.7	108
Education					
	No education	53.5	797	52.9	533
	1–4 years	13.2	196	14.6	147
	5–9 years	22.4	334	20.9	211
	Secondary or greater	10.5	157	11.6	117
Works for pay		44.6	664	48.8	493
Has had intercourse		85.6	1276	92.5	922
Age at 1st birth					
	Has not given birth	19.7	294	14.2	141
	<17 years	24.4	363	26.0	258
	17–19 years	32.3	481	37.4	371
	>19 years	17.8	265	18.8	186
Number of living children					
	0	20.7	309	14.8	149
	1–2	28.7	428	32.5	327
	3–4	24.3	362	26.3	265
	5+	25.6	382	26.4	266
Ideal number of children					
	0	14.2	211	11.8	117
	1–2	7.9	118	8.3	82
	3–4	35.4	528	38.1	379
	5+	39.8	593	41.9	416
Agree with husband/partner about ideal number of children					
	Agree	41.7	622	49.6	502
	Disagree	16.9	252	18.4	186
	Don't know/not with partner	41.1	613	32.1	325

* Denominator in percent calculations include missing responses.

**Table 2:** Injectable contraceptive factors among all women surveyed and among the WTP study subpopulation (i.e. women who had ever used injectable contraceptives or expressed interest in using them).

		Full Sample N = 1490*		Subpopulation N = 1013	
		%	N	%	N
Injectable contraceptive is preferred method of contraception		55.3	824	76.8	730
Has ever used injectable contraceptives		46.2	688	67.0	665
Currently using injectable contraceptives		20.6	307	32.8	303
Length of time using injectable contraceptives					
	Never used	49.3	732	33.7	328
	<1 year	14.6	218	22.1	215
	1–2 years	14.7	219	21.7	211
	>2 years	14.9	222	22.4	218
Knows correct coverage time of injectable contraceptives		78.1	1163	92.6	884

* Denominator in percent calculations include missing responses.

**Table 3:** Structural factors among all women surveyed and among the WTP study subpopulation (i.e. women who had ever used injectable contraceptives or expressed interest in using them).

		Full Sample N = 1490*		Subpopulation N = 1013	
		%	N	%	N
Time to facility					
	<30 minutes	44.6	665	46.0	462
	30+ minutes	54.5	812	54.0	543
Whether visited health facility and received family planning in last 12 months					
	Didn't visit	26.9	400	20.0	201
	Visited and didn't receive	12.4	185	14.1	141
	Visited and received	59.5	887	65.9	661
Exposed to family planning messages on TV/magazine/newspaper in last few months		38.1	567	42.4	415

* Denominator in percent calculations include missing responses.

Table 4 displays the bivariate results of the chi-squared tests investigating the percent willing to pay for injectable contraceptives among each covariate, which were used to determine which variables were included in the model. Among the individual level factors, age, marital status, education, payment for work, whether has had sex, age at first birth, and number of living children were all significantly associated with WTP at the p≤0.05 level. Among the injectable contraceptive factors, preferred method of contraception, ever-use of injectable contraceptives, average time using injectable contraceptives, and whether knows correct coverage time of injectable contraceptives were significantly associated with WTP at the p≤0.05 level. The structural factors that were significant at the p≤0.05 level include time to facility and health facility visit in the last 12 months (regardless of receipt of family planning method).

**Table 4:** Characteristics of the subpopulation by percent willing to pay for injectable contraceptives (N = 1013).

		% willing to pay	N	p-value
Age				
	15–19	76.2	151	<0.001
	20–24	75.5	200	
	25–29	70.9	227	
	30–34	67.4	184	
	35–39	64.8	139	
	40–44	50.8	65	
	45–49	40.0	35	
Marital status				
	Never married	81.0	84	0.004
	Married/cohabiting	68.4	820	
	Divorced/widowed	58.3	108	
Education				
	No education	61.4	533	<0.001
	1–4 years	66.0	147	
	5–9 years	77.7	211	
	Secondary or greater	87.2	117	
Works for pay				
	No	59.8	517	<0.001
	Yes	77.3	493	
Has had sex				
	No	85.3	75	0.001
	Yes	67.3	922	
Age at 1st birth				
	Has not given birth	80.9	141	<0.001
	<17 years	65.5	258	
	17–19 years	70.4	371	
	>19 years	67.2	186	
	Don't know	25.7	35	
Number of living children				
	0	81.2	149	<0.001
	1–2	71.3	327	
	3–4	65.7	265	
	5+	60.2	266	
Ideal number of children				
	0	70.1	117	0.056
	1–2	65.9	82	
	3–4	72.6	379	
	5+	63.7	416	
Agree with husband/partner about ideal number of children				
	Agree	70.1	502	0.422
	Disagree	65.1	186	
	Don't know/not with partner	67.7	325	
Preferred method of contraception				
	Not injectable contraceptives	57.5	221	<0.001
	Injectable contraceptives	73.7	729	
Has ever used injectable contraceptives				
	No	78.4	328	<0.001
	Yes	63.9	664	
Currently using injectable contraceptives				
	No	65.9	621	0.345
	Yes	69.0	303	
Length of time using injectable contraceptives				
	Never used	78.1	319	<0.001
	<1 year	57.6	217	
	1–2 years	62.9	213	
	>2 years	71.0	221	
Knows correct coverage time of injectable contraceptives				
	No	80.3	71	0.046
	Yes	69.0	884	
Time to facility				
	<30 minutes	73.8	462	0.001
	30+ minutes	63.9	543	
Whether visited health facility and received family planning in last 12 months				
	Didn't visit	52.7	201	<0.001
	Visited and didn't receive	80.1	141	
	Visited and received	71.3	661	
Exposed to family planning messages on TV/magazine/newspaper in last few months				
	No	68.6	563	0.40
	Yes	71.1	415	

In our final logistic regression model, several factors were found to be significantly associated with WTP, including both sets of cross-products (Table 5). Among the factors not associated with the interaction terms, being married (OR = 4.54, 95% CI 1.01, 20.48), all levels of education (1–4 years OR = 1.71, 95% CI 1.03, 2.85; 5–9 years OR = 1.92, 95% CI 1.08, 3.43; secondary school or higher OR = 3.51, 95% CI 1.64, 7.51), age at first birth (less than 17 years OR = 2.85, 95% CI 1.47, 5.53; 17–19 years OR = 3.05, 95% CI 1.30, 7.14; and greater than 19 years OR = 2.92, 95% CI 1.26, 6.78) and having visited a health facility in the last 12 months (whether respondent received family planning information or not, OR = 3.07, 95% CI 1.58, 5.95 and OR = 3.66, 95% CI 1.44, 9.32, respectively) were associated with statistically significantly increased odds of WTP. Having initiated sexual activity (OR = 0.17, 95% CI 0.03, 0.97), having 1–2 children (OR = 0.33, 95% CI 0.18, 0.61), and having used injectable contraceptives for any amount of time when they were not the preferred method (OR = 0.13, 95% CI 0.07, 0.27; OR = 0.20, 95% CI 0.11, 0.38; OR = 0.23, 95% CI 0.08, 0.69 for use less than1 year, 1 to 2 years, and great than 2 years, respectively) were associated with statistically significantly decreased odds of WTP.

**Table 5:** Final logistic regression model investigating willingness to pay for injectable contraceptives among women who have ever used injectable contraceptives or who expressed interest in using injectable contraceptives when asked (N = 849).

		OR	p-value	95% CI
Age		0.98	0.263	0.946, 1.014
Marital status				
	Never married	–	–	Reference
	Married/cohabiting	4.54	0.049	1.008, 20.479
	Divorced/widowed	3.08	0.147	0.673, 14.125
Education				
	No education	–	–	Reference
	1–4 years	1.71	0.040	1.025, 2.847
	5–9 years	1.92	0.026	1.081, 3.426
	Secondary or greater	3.51	0.001	1.638, 7.514
Works for pay				
	No	–	–	Reference
	Yes	4.07	<0.001	2.302, 7.194
Has had sex				
	No	–	–	Reference
	Yes	0.17	0.047	0.029, 0.973
Age at first birth				
	Has not given birth	–	–	Reference
	<17 years	2.85	0.002	1.468, 5.526
	17–19 years	3.05	0.010	1.300, 7.137
	>19 years	2.92	0.013	1.256, 6.784
	Don't know	0.33	0.130	0.081, 1.380
Number of living children				
	0	–	–	Reference
	1–2	0.33	<0.001	0.182, 0.609
	3–4	0.38	0.141	0.106, 1.374
	5+	0.41	0.141	0.125, 1.343
Agree with husband/partner about ideal number of children				
	Agree	–	–	Reference
	Disagree	1.26	0.455	0.684, 2.335
	Don't know/not with partner	1.85	0.205	0.714, 4.799
Preferred method of contraception				
	Not injectable contraceptives	–	–	Reference
	Injectable contraceptives	0.82	0.576	0.399, 1.666
Length of time using injectable contraceptives				
	Never used	–	–	Reference
	<1 year	0.13	<0.001	0.065, 0.266
	1–2 years	0.20	<0.001	0.107, 0.384
	>2 years	0.23	0.009	0.079, 0.694
Whether visited health facility and received family planning in last 12 months				
	Didn't visit	–	–	Reference
	Visited and didn't receive	3.66	0.007	1.437, 9.318
	Visited and received	3.07	0.001	1.578, 5.952
Payment for work and agree with husband about ideal number of children cross-products				
	Not paid	–	–	Reference
	Paid and disagree with husband/partner	0.84	0.631	0.411, 1.714
	Paid and don't know husband's/partner's preference/not with partner	0.30	0.003	0.131, 0.668
Preference for injectable contraceptives and length of time have used it				
	Don't prefer	–	–	Reference
	Prefer injectables and have used <1 year	3.64	<0.001	1.798, 7.365
	Prefer injectables and have used 1–2 years	3.67	0.002	1.604, 8.402
	Prefer injectables and have used >2 years	6.25	0.007	1.655, 23.602

The odds of WTP for injectable contraceptives among women who work for pay compared to those who do not work for pay vary by whether they agree with their husband/partner’s ideal number of children (Table 6). Women who disagree with their husband/partner’s ideal number of children and who work for pay have 3.42 times the odds of being willing to pay (95% CI 2.01, 5.62) compared to women who disagree and do not work for pay. Women who do not know their husband/partner’s ideal number of children or who are not with a partner and who work for pay have 1.21 times the odds of being willing to pay (95% CI 0.59, 2.45) compared to women who do not know or are not with a partner and do not work for pay. And women who agree with their husband/partner’s ideal number of children and who work for pay have 4.07 times the odds of being willing to pay (95% CI 2.30, 7.19) compared to women who agree and do not work for pay.

**Table 6:** Lincom results for odds ratios of interactions (N = 849).

	OR	p-value	95% CI
**Odds ratios of WTP by works for pay and agreement with husband/partner about ideal number of children**			
Disagree: paid vs not paid	3.42	*<0.001	2.077, 5.619
Don't know: paid vs not paid	1.21	0.605	0.594, 2.445
Agree: paid vs not paid	4.07	*<0.001	2.302, 7.194
**Odds ratios of WTP by preference for injectable contraceptives and length of time have used it**			
Have never used: prefer vs don't prefer	0.82	0.576	0.400, 1.666
Have used <1 year: prefer vs don't prefer	2.97	*<0.001	1.834, 4.803
Have used 1–2 years: prefer vs don't prefer	2.99	*<0.001	1.924, 4.659
Have used >2 years: prefer vs don't prefer	5.10	*0.001	1.902, 13.660

P-values and 95% CIs presented are lincom results before adjustment.

* Indicates statistical significance after Bonferroni adjustment.

The odds of WTP for injectable contraceptives among women who prefer injectable contraceptives compared to those who do not prefer injectable contraceptives vary by length of time using injectable contraceptives (Table 6). Women who have never used injectable contraceptives and who prefer them to other methods of contraception have 0.82 times the odds of being willing to pay (95% CI 0.40, 1.67) compared to those who do not prefer them. Women who have used injectable contraceptives for less than one year and who prefer them have 2.97 times the odds of being willing to pay (95% CI 1.83, 4.80) compared to those who do not prefer them. Women who have used injectable contraceptives for one to two years and who prefer them have 2.99 times the odds of being willing to pay (95% CI 1.92, 4.66) compared to those who do not prefer them. And women who have used injectable contraceptives for more than two years and who prefer them have 5.10 the odds of being willing to pay (95% CI 1.90, 13.66) compared to those who do not prefer them.

## Discussion

To our knowledge this is the first study to assess factors associated with WTP for injectable contraceptives. Results from the multivariate analyses revealed multiple individual level, injectable contraceptive, and structural factors to be associated with a woman’s WTP for injectable contraceptives.

Among individual level factors, increasing level of education was associated with greater WTP for injectable contraceptives. This finding may reflect the strong associations between women’s education and contraceptive use found in previous studies conducted in Ethiopia [22], [23]. In a study investigating WTP for insecticide treated bed nets in southern Ethiopia, researchers also found that education was positively associated with WTP [24].

Initiation of sexual activity was another significant individual level factor. Survey respondents who had not yet initiated sexual activity had a statistically significantly higher WTP for injectable contraceptives than their sexually active counterparts. This may indicate that they are more motivated to control their fertility even before the initiation of sexual activity. These respondents are younger and have a lower average desired fertility (data not shown), and they are likely better informed. This younger generation’s higher WTP bodes well for future cost recovery, as the expectations of these women may differ from the previous generations’, whose only experience with reproductive health care in rural areas has been free government services and commodities. Another interpretation of this finding is that WTP for contraception among non-sexually active women diminishes once they become sexually active and the question is no longer hypothetical.

We also detected a statistically significant association between WTP and age at first birth, another individual level factor. Regardless of the age at first birth, women who have given birth appear more motivated to use contraception as a means of controlling their fertility than those who have not given birth.

We found an interaction between working for pay and agreement with husband/partner’s ideal number of children, both of which are individual level factors. This indicates a strong connection between SES (or in our case a proxy for SES), motivation for achieving desired family size, and WTP for injectable contraceptives. This finding is in line with previous research [4], [12]. Those who receive payment for work had significantly increased odds of WTP compared to those who do not receive payment. The level of increased odds among those who receive payment was dependent on whether or not they agree with their husband/partner’s ideal number of children, with those who agree having the highest odds. This perhaps indicates that these women are particularly motivated to achieve their desired family size and they feel comfortable using money to do so since their husband/partner similarly wants to achieve that family size.

As could be expected, the injectable contraceptive factor of preference for injectable contraceptives was associated with increased odds of WTP for injectable contraceptives, but only if the woman had actually used the method. Preferring injectable contraceptives and having used this method for any amount of time was associated with significantly increased odds of WTP compared to those women who did not prefer it. If WTP is considered a reflection of demand [12] and we assume that women who prefer injectable contraceptives have a greater demand for this method, it is logical that they would be more motivated to use injectable contraceptives and thus more willing to pay for them. This logic follows previous findings in the literature. Foreit and Foreit (2003) examined WTP for contraceptive pills and found that women whose first choice of contraception was pills were more willing to pay for them than women whose first choice was not pills. The authors concluded that women who preferred pills were more motivated to use this method and therefore were more willing to pay [4]. WTP’s relationship to demand is discussed further below.

The final model also indicates that having used injectable contraceptives for any amount of time and not preferring this method is associated with significantly decreased odds of WTP for it. This interaction between two injectable contraceptive factors seems counterintuitive, until considering that currently, the only source of injectable contraceptives in rural areas is the government, which provides them for free. If respondents have previously received injectable contraceptives for free from government facilities, their expectation will be that injectable contraceptives are a commodity that should not require payment [12], [24], [25]. Existing literature indicates that expectations are integral in shaping women’s WTP for a health commodity or service [24], [25]. In addition, because it is not their preferred method, their motivation to use it (and pay for it) is likely to be lower than those who prefer it. As a result, women who had been using injectable contraceptives for any amount of time but did not prefer the method were less willing to pay for the services, demonstrating the interplay of expectations and motivation.

Among the structural factors, women who visited a health facility in the last 12 months (whether they received family planning information or not) had significantly increased odds of WTP. It is important to note that this correlation does not necessarily indicate that a causal relationship exists, because women who visit health facilities might also differ in other ways, such as attitude towards contraception and education level. This is an interesting result given that the health facilities in the surveyed communities are public and provide free family planning services. Qualitative findings from Malawi offer an explanation for this finding, suggesting that even in settings where free government services arethe norm, there are a range of factors influencing WTP (i.e. method stock-outs, transport, or other hidden costs) [5]. Alternatively, this finding could be explained through motivation [4], in that women who have visited a health facility in the last 12 months are more motivated to use contraception than those who have not.

Although this analysis revealed many new and interesting findings, they should be taken in the context of the paper’s limitations. Unfortunately, we did not have a variable that allowed us to determine a household’s socioeconomic status in terms of income or assets. Much of the previous literature found this measure of SES to be strongly associated with WTP [4], [12]. We used whether a woman is paid for her work (in cash, in kind, or both) as a proxy for economic status, but it is admittedly insufficient. As a result, there is likely unaccounted-for confounding. Also controlling for education likely improves our proxy for SES though. Our variable related to preferred method of contraception (injectable versus not injectable) is from a survey question that many respondents had difficulty answering because there is no word for ‘preference’ in the local language. This may have compromised the validity of responses to the question. We included only women interested in using injectable contraceptives in this analysis, which likely positively biased the results since women not interested in using the method are presumably less willing to pay for it. With regards to the WTP questions, previous studies have found variations in results based on the elicitation method applied. Thus our results may have been different had we used the ‘bidding game’ method (an iterative process where a respondent is asked whether they are willing to pay a given amount and the follow up question asks about a higher or lower amount depending upon the initial response) or the ‘take-it-or-leave-it’ method (where the amount asked varies across surveys and the question is only asked once of each respondent) [26]. In addition, it is important to note that the WTP questions referred to whether respondents were willing to pay for injectable contraceptives generally, not in the context of the improved convenience and confidentiality provided by CBD. Previous literature has demonstrated that consumers are more likely to pay new or increased fees if they are paired with improved quality or less travel time. If we consider this, the proportion of women willing to pay for CBD of injectable contraceptives in this population is likely even higher [5], [13], [18], [27], [28]. It is important to distinguish between WTP and ability to pay; the two concepts are different and WTP is not a perfect predictor of demand. As indicated by the WTP-based demand curve, of those women who are willing to pay, the amount for many is quite small and subsidization would be necessary in Ethiopia. Despite these limitations, the results from our analyses offer insight into the individual level, injectable contraceptive, and structural factors that are associated with a woman’s WTP for injectable contraceptives in Tigray, Ethiopia. Findings allow government and non-profit healthcare organizations to begin understanding ways they can improve cost recovery for injectable contraceptive provision.

In general, it is also important to consider the broader context in which the project and associated user fee is being implemented. In rural Tigray, Ethiopia, the only source of injectable contraception is government facilities, which are subject to stock outs and can be quite far for the most rural women. Although some women may not be able or willing to pay for injectable contraceptives from a CBRHA in the project, the overall burden of unplanned pregnancies would still likely be diminished because this project only adds to existing injectable contraceptive access points; women who cannot pay for the convenience and confidentiality of this service can still receive free injections from existing government facilities. Additional research should be done to determine whether adding a user fee without increasing access increases or decreases the overall cost to the health care system when factoring in unplanned pregnancies.

Cost recovery for family planning services may offer a means of improved financial sustainability in a sector that continually struggles with funding while increasing rural access to injectable contraceptives, the preferred method of contraception in Ethiopia. This study demonstrates that there is substantial WTP for injectable contraceptives and provides insight into which factors are associated with WTP among women in Tigray, Ethiopia. Preference for injectable contraceptives is highly associated with WTP. Preference and motivation can likely be influenced by information, education, and communication campaigns and family planning counseling that highlight the importance of contraception [29], [30], [31]. Educating women on modern methods of contraception and helping them determine their preferred method of contraception could be a means of increasing their demand/motivation and WTP. An important consideration is the quality of services. Researchers have repeatedly demonstrated that improved quality of services and/or access is positively correlated with increased WTP [5], [13], [18], [27], [28]. Government and private sector health care systems should keep this in mind when considering implementing or increasing user fees.

## Conclusions

This study contributes to the literature examining WTP for contraceptives and is the first to investigate factors associated with WTP for injectable contraceptives specifically. Ethiopian women are not alone in their preference for injectable contraceptives [32]. This method is widely preferred among women in sub-Saharan Africa, with an estimated 9 million users constituting 43% of total contraceptive use in the region [33]. More research is needed to better understand what contributes to women’s WTP so that government and private sector health care systems can work to maximize WTP and ensure that those unwilling or unable to pay still have access to the necessary services. Health equity is of utmost importance, but sustainability is of growing concern and achieving even partial cost recovery will be an increasingly important aspect of health care delivery in the future.
